# In Silico Evaluation of the Haplotype Diversity, Phylogenetic Variation and Population Structure of Human *E. granulosus sensu stricto* (G1 Genotype) Sequences

**DOI:** 10.3390/pathogens11111346

**Published:** 2022-11-14

**Authors:** Muhammed Ahmed Selcuk, Figen Celik, Harun Kaya Kesik, Seyma Gunyakti Kilinc, Haroon Ahmed, Nan Jiang, Sami Simsek, Jianping Cao

**Affiliations:** 1 Department of Parasitology, Faculty of Veterinary Medicine, Siirt University, Siirt 56100, Turkey; 2 Department of Parasitology, Faculty of Veterinary Medicine, University of Firat, Elazig 23119, Turkey; 3 Department of Parasitology, Faculty of Veterinary Medicine, Bingol University, Bingol 12000, Turkey; 4 Department of Biosciences, COMSATS University Islamabad (CUI), Park Road, ChakhShazad, Islamabad 45550, Pakistan; 5 National Institute of Parasitic Diseases, Chinese Center for Disease Control and Prevention, (Chinese Center for Tropical Diseases Research), Shanghai 200025, China; 6 Key Laboratory of Parasite and Vector Biology, National Health Commission of the People’s Republic of China, Shanghai 200025, China; 7 World Health Organization Collaborating Center for Tropical Diseases, Shanghai 200025, China; 8 The School of Global Health, Chinese Center for Tropical Diseases Research, Shanghai Jiao Tong University School of Medicine, Shanghai 200240, China

**Keywords:** *Echinococcus granulosus s.s.*, G1 genotype, CO1, ND1, genetic variability

## Abstract

*Echinococcus granulosus sensu lato* is the causative agent of cystic echinococcosis (CE), which is a neglected zoonotic disease with an important role in human morbidity. In this study, we aimed to investigate the haplotype diversity, genetic variation, population structure and phylogeny of human *E. granulosus sensu stricto* (s.s.) (G1 genotype) isolates submitted to GenBank from different parts of the world by sequencing the mitochondrial CO1 and ND1 genes. The sequences of the mt-CO1 (401 bp; *n* = 133) and mt-ND1 (407 bp; *n* = 140) genes were used to analyze the haplotype, polymorphism and phylogenetic of 273 *E. granulosus s.s.* (G1 genotype) isolates. Mutations were observed at 31 different points in the mt-CO1 gene sequences and at 100 different points in the mt-ND1 gene sequences. Furthermore, 34 haplotypes of the mt-CO1 sequences and 37 haplotypes of the mt-ND1 sequences were identified. Tajima’s D, Fu’s Fs, and Fu’s LD values showed high negative values in both mt-CO1 and mt-ND1 gene fragments. The haplotype diversities in the sequences retrieved from GenBank in this study indicate that the genetic variation in human isolates of *E. granulosus s.s.* in western countries is higher than in eastern countries. This may be due to demographic expansions due to animal trades and natural selections.

## 1. Introduction

Cystic echinococcosis (CE) is a neglected parasitic zoonotic disease caused by *Echinococcus granulosus sensu lato* and has an important role in human morbidity. CE is distributed worldwide, especially in Asia, Africa, Europe, South America, Canada and Australia [1,2,3]. In 2014, CE was ranked as the third most important foodborne parasitic disease globally by the Food and Agriculture Organization (FAO) and World Health Organization (WHO), and *E. granulosus s.l.* infections are responsible for billions of dollars of economic loss per year [4,5,6].

In recent years, according to the mitochondrial cytochrome c oxidase subunit 1 (mt-CO1) and NADH dehydrogenase subunit 1 (mt-ND1) DNA sequences, the genetic diversity of *E. granulosus s.l.* was reported. These were designated *E. granulosus sensu stricto* (s.s.) (G1 and G3), *E. equinus* (G4), *E. ortleppi* (G5) and *E. canadensis* (G6/7, G8-10), and *E. felidis* [7,8,9]. However, *E. granulosus s.s.* is the most common of these, constituting the majority of human CE infections (approximately 89%), and also has the most zoonotic features [10,11].

Two interrelated hosts play an important role in the life cycle of *E. granulosus s.l*. Carnivores, mostly dogs, are final hosts, and many mammals, including humans, are intermediate hosts. Accidental ingestion of eggs from the feces of infected host species leads to the infection of different internal organs of the intermediate host, but mainly the liver and lungs [12,13,14].

Although CE is a benign disease, it can progress with high morbidity and mortality as a result of unexpected and serious complications [15]. However, the clinical symptoms vary based on the size, location and condition of the cyst. After ingestion, the embryos are released from the eggs into the small intestine, where they penetrate the mucous membranes, mix with the blood and reach many organs. Although a single cyst is prevalent in the majority of infected organisms, multiple cysts or cyst formation in multiple organs can be observed in 20–40% of individuals. Most cysts occur in the liver (>65%), followed by the lung (25%), while they are less common in the spleen, kidney, bone, heart and central nervous system [16,17].

Human CE is widely distributed throughout the world. The prevalence of surgically managed human CE per 100,000 has been reported at 32 in Central and Southern Peru [18], 6–20 in Southwest Chile in 2005 [19], 30 in Argentina Rio Negro [20], 1.5 in Northern Israel [21], 0.68 in Southern Israel [21], 80 in China-Xinjiang [22], 4.2 in Eastern Libya [23], 1.3–2.6 in Egypt [24], 15 in Tunisia [25], 3.6–4.6 in Algeria [26], 10.8 in Spain-Salamanca between 1980–2000 [27], 10 in France-Corsica [28], 1.3 in Italy [29] and 4.55 in Morocco in 2006 [30]. Its prevalence was also reported at between 3.5% and 6% in urban and rural areas of Brazil [31].

Communities in which sheep breeding is widespread contribute greatly to this distribution, with *E. granulosus s.s.* (G1 genotype) playing an important role in transmission in humans [32,33,34].

Due to the maternal inheritance and high mutation rates of mitochondrial (mt) DNA sequences, these sequences are commonly analyzed to determine the genetic structure of the population and the degree of close kinship [35]. In many studies, partial sequences of mt-CO1 and mt-ND1 genes have been used successfully to distinguish genetic variants among *Echinococcus* species and between *E. granulosus* strains [36,37,38].

Global evaluation of genetic variation among human isolates of *E. granulosus s.s.* (G1 genotype) is important to reveal the population dynamics of the parasite. In the current study, we evaluated the haplotype diversity, genetic variation, population structure and phylogeny of human *E. granulosus s.s.* (G1 genotype) submitted to GenBank from different parts of the world by analyzing the mt-CO1 and mt-ND1 gene sequences.

## 2. Materials and Methods

### 2.1. Data Collection

After filtering the mt-CO1 (*n* = 382) and mt-ND1 (*n* = 199) gene sequences containing human (*Homo sapiens*) isolates of the *E. granulosus s.s.* (G1 genotype) submitted to the National Center for Biotechnology Information, USA, (NCBI) (www.ncbi.nlm.nih.gov) database until 6 April 2022, a total of 581 gene sequences were obtained and a dataset was created.

### 2.2. Alignment and Phylogenetic Analysis

All the gene sequences were loaded into the CLC Sequence Viewer 8 [39] in FASTA format. All sequences were trimmed from both ends and were then aligned using the mt-CO1 (accession no. MG672129) and mt-ND1 (accession no. KU925413) reference sequences. After the removal of short gene sequences, the remaining 273 gene sequences [401 bp mt-CO1 (*n* = 133) and 407 bp mt-ND1 (*n* = 140)] were used for bioinformatic analysis. Individual phylogenetic trees were created from the sequences of both gene regions using the neighbor-joining (NJ) model and the Jukes-Cantor nucleotide distance measure. Statistical support for the specificity of the branches was obtained using 1000 bootstrap replicates. *Taenia saginata* and *T. solium* sequences were added as outgroups to show the degree of relations.

### 2.3. Haplotype Analysis and Networking

The haplotype analysis was carried out using the DnaSP 6 program in which the sequences were investigated in FASTA format [40]. The haplotype and nucleotide change values, nucleotide and haplotype numbers and neutrality indexes were calculated to determine the genetic structure of both gene regions. The sequences were converted to Nexus format [41] and a haplotype network was generated by using the PopArt (Population Analysis with Reticulate Trees) program [42] for a visual representation of the relationships between haplotypes.

## 3. Results

In this study, we analyzed a total of 273 gene sequences of *E. granulosus s.s.* (G1 genotype) isolates obtained from the NCBI database, consisting of 133 mt-CO1 sequences from 15 countries and 140 mt-ND1 sequences from 16 countries (Table 1).

**Figure 1:**
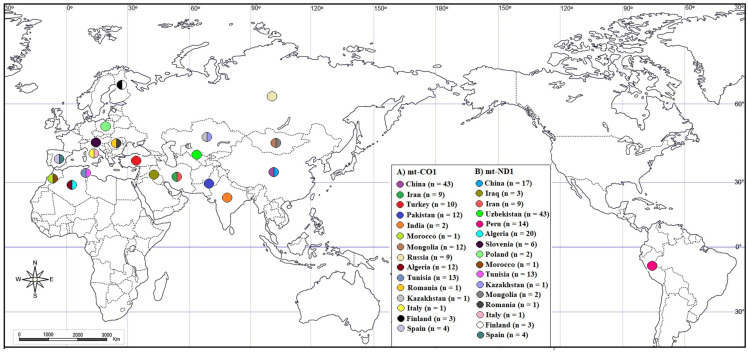
Distribution map of mt-CO1 and mt-ND1 sequences of *E. granulosus s.s.* (G1 genotype) by geographical regions.

The distribution of the collected mt-CO1 and mt-ND1 sequences over the world is shown in Figure 1.

### 3.1. Polymorphism and Haplotype Analysis

Mutations were observed at 31 different points within the mt-CO1 gene sequences, with the longest conserved areas detected between 116 bp and 167 bp. Within the mt-ND1 sequences, mutations were observed at 100 different points, with conserved areas detected between 361 bp and 407 bp. No protein-coding domain was found in either of the datasets. Analysis of 133 mt-CO1 gene sequences revealed 34 different haplotypes (Table 2). Among these, Hap03 constituted the main haplotype with 79 gene sequences, of which 23 existed as a single haplotype. Analysis of 140 mt-ND1 gene sequences revealed 37 haplotypes (Table 3). Among these, Hap01 constituted the main haplotype, with 83 gene sequences, constitutes of which 28 existed as a single haplotype.

### 3.2. Haplotype Network

**Figure 2:**
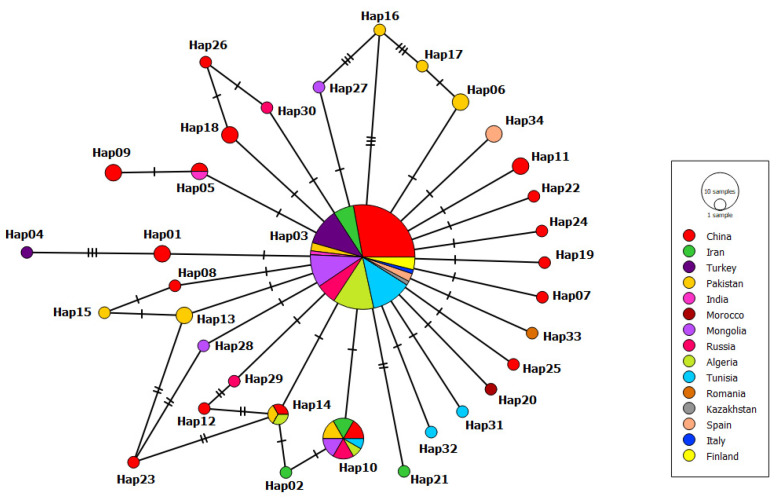
Appearance of mt-CO1 (401 bp) haplotypes *E. granulosus s.s.* (G1 genotype) sequences. The number of mutations that distinguish haplotypes is indicated by screening marks. The geographical distribution of haplotypes is shown in different colors. The size of the circles is related to the haplotype frequency.

The mt-CO1 haplotype network consisted of 34 haplotypes (Figure 2). A comparison of the main haplotype with the others in this network revealed between one and seven mutations. The main haplotype was Hap03, accounting for 59.39% (79/133) of the haplotype network, followed by Hap10, accounting for 9.02%. (12/133). A unique single haplotype constituted 67.64% (23/34) of the haplotype network. Single haplotypes were from China (*n* = 9), Pakistan (*n* = 3), Iran (*n* = 2), Tunisia (*n* = 2), Russia (*n* = 2), Mongolia (*n* = 2), Turkey (*n* = 1), Romania (*n* = 1) and Morocco (*n* = 1).

**Figure 3:**
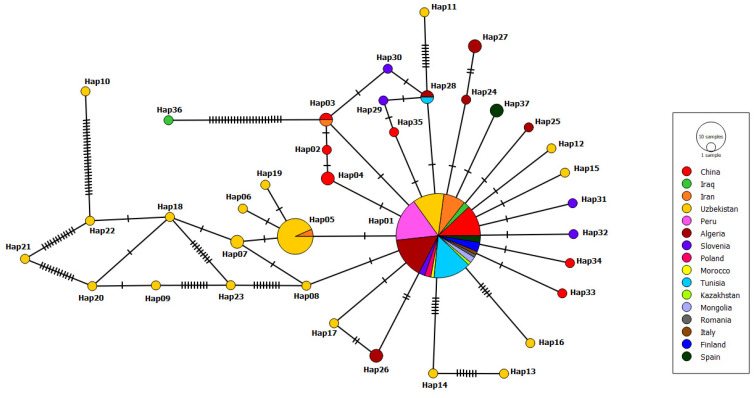
Appearance of mt-ND1 (407 bp) haplotypes of *E. granulosus s.s.* (G1 genotype) sequences. The number of mutations that distinguish haplotypes is indicated by screening marks. The geographical distribution of haplotypes is shown in different colors. The size of the circles is related to the haplotype frequency.

The mt-ND1 haplotype network consisted of 37 haplotypes (Figure 3). A comparison of the main haplotype with the others in this network revealed between one and 50 mutations. The main haplotype was Hap01, accounting for 59.28% (83/140) of the haplotype network, followed by Hap05, accounting for 10.71% (15/140). A unique single haplotype constituted 75.67% (28/37) of the haplotype network. Single haplotypes were from Uzbekistan (*n* = 17), Slovenia (*n* = 4), China (*n* = 4), Algeria (*n* = 2) and Iraq (*n* = 1).

The nucleotide positions of the mt-CO1 and mt-ND1 genes among the haplotypes were presented in Supplementary Tables S1 and S2.

### 3.3. Phylogenetic Tree

**Figure 4:**
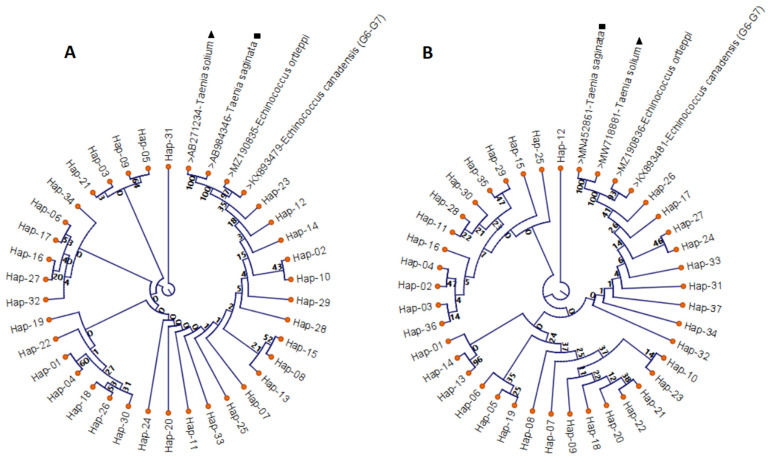
Phylogenetic tree view of *E. granulosus s.s.* (G1 genotype) sequences using mt-CO1 (401 bp) (**A**) and mt-ND1 (407 bp) (**B**) gene and reference sequences. CLC Sequence Viewer 8 was used to generate a Maximum Likelihood tree based on the Neighbor Joining model. The reliability of the tree was evaluated with 1000 bootstrap iterations. ■ *Taenia saginata ▲ Taenia solium*.

The results of the phylogenetic analysis were consistent with the haplotype network. The phylogenetic tree generated by aligning the mt-CO1 gene sequences is shown in Figure 4A. In this tree, Hap04 (EU006783), Hap12 (DQ356874), Hap16 (MK229315) and Hap23 (AB688619) were the haplotypes farthest apart, with mutations at seven points. The phylogenetic tree generated by aligning the mt-ND1 gene sequences is shown in Figure 4B. In this tree, Hap10 (MN696602) and Hap36 (MN231833) were the haplotypes farthest apart, with mutations at 50 points. *Taenia saginata* and *T. solium* were added as outgroups in both phylogenetic trees.

### 3.4. Gene Flow, Diversity and Neutrality Analysis

The diversity and neutrality indices of the mt-CO1 and mt-ND1 groups are shown in Table 4. Tajima’s D (Tajima, 1989) and Fu’s FS (Fu, 1997) values were calculated to determine whether populations were subject to selection pressure. Tajima D, Fu’s Fs and Fu’s LD values showed high negative values in both the mt-CO1 and mt-ND1 regions, providing evidence of a large number of alleles.

## 4. Discussion

Genetic diversity and population structure of *E. granulosus s.s.* (G1 genotype) were investigated in the current study. This was carried out using sequenced data of mt-CO1 and mt-ND1 retrieved from GenBank, commonly used for the differentiation of Echinococcus species. Results obtained in this study emerged information about gene flow and population dynamics in human *E. granulosus s.s.* infections globally. A total of 133 mt-CO1 (401 bp) and 140 mt-ND1 (407 bp) gene sequences of *E. granulosus s.s.* (G1 genotype) human isolates already registered in the NCBI database were used for us in silico analyses to determine the genetic diversity and variations of the *E. granulosus s.s.* (G1 genotype) human isolates.

Although the prevalence and incidence of CE have decreased significantly in recent years, it still remains an important public health concern, especially in some countries and geographical regions that cannot implement a control program due to economic difficulties [43]. In addition, it is an important problem for human health in developing countries where animal husbandry is intense, and sheep meat is consumed intensively [44]. The incidence of CE increases with age and is more common between the ages of 20 and 40 years. The incidence of the disease is higher in societies with a low socio-economic ratio [45]. 

The results of the current study show an extremely high global haplotype diversity within the G1 genotype. The 273 samples analyzed represented a total of 34 haplotypes for mt-CO1 and 37 for mt-ND1. High genetic diversity within *E. granulosus s.s.* has also been reported by Kinkar et al. [46]. They [46] analyzed 212 samples (near complete mitochondrial sequence) and found 171 haplotypes (overall haplotype diversity was 0.994). The main reason for the haplotype difference between the studies is related to the length of the gene regions analyzed. Therefore, more haplotypes can be determined by sequencing longer mitochondrial gene fragments.

Neutrality indices such as Tajima D, Fu’s Fs, and Fu’s LD were used to measure nucleotide variability and population expansion [47]. The Tajima D test evaluates the deviation of populations from the standard neutral model, with a positive Tajima D value representing heterozygosity, defined as having a selective advantage, while negative values indicate that a particular allele has a selective advantage over the other allele. A negative value also indicates a rapid increase in the population [48,49]. In our study, Tajima D values were low in both the mt-CO1 and mt-ND1 gene fragments, indicating a high probability of population increase in the future. However, the lower Tajima D value of the mt-ND1 gene sequence (−2.80355) compared with that of the mt-CO1 gene sequence (−2.47269) indicates a higher rate of population growth in the former. The negative value of the neutrality indices Tajima’s D suggests population expansion (Animal movements among the countries indicate that this expansion may continue in the coming years. Fu’s FS represents a marker of sensitivity to population growth, with a significantly negative value (*p* < 0.05), indicating that the populations have common growth patterns and belong to the same gene pool [50,51]. Our analysis yielded highly negative and statistically significant Fu’s Fs values in both the mt-CO1 and mt-ND1 haplotype groups, indicating that these populations are subject to expansion globally.

Nucleotide diversity was examined to determine the degree of polymorphism in the population. We determined that the mean nucleotide difference of the mt-ND1 (0.00611) gene sequence was higher than that of the mt-CO1 (0.00255) gene sequence. In addition, haplotype diversity was assessed to evaluate the uniqueness of haplotypes within the population. In our study, the values of the mt-CO1 (0.640) and mt-ND1 (0.639) gene sequences were very similar.

In total, 34 haplotypes were identified in our analysis of the mt-CO1 gene sequences. The main haplotype constituted 59.39% of the total network, and there were 23 single haplotypes. Thirty-seven different haplotypes were identified in our analysis of the mt-ND1 gene sequence. The main haplotype constituted 59.28% of the total network, and there were 28 single haplotypes. The major haplotypes represent a single ancestor. 

In total, 31 different mutations were detected across the 401 bp mt-CO1 gene sequences, and 100 different mutations were detected within the 407 bp mt-ND1 sequences. The higher mutation rates may reflect the long and complex evolutionary history of *E. granulosus*. The genetic diversity within *E. granulosus s.s.* (G1 genotype) is very high worldwide, and the observed complex phylogeographic patterns emerging from the phylogenetic and geographic analyses suggest that the current distribution of *E. granulosus s.s.* (G1 genotype) has been shaped by the intensive animal trade [46]. The high number of haplotypes detected in some Asian and Middle Eastern countries (China, Mongolia, Pakistan, Iran, etc.) in this study may indicate that *E. granulosus s.s*. (G1 genotype) has existed in these countries for many years compared to some western countries (Finland and Spain).

## 5. Conclusions

*E. granulosus s.s.* (G1 genotype) poses an important problem in communities where sheep breeding is common. Although different molecular studies have been conducted to date, this study is the first bioinformatics study to evaluate the genetic structure and gene flow of human isolates of the *E. granulosus s.s.* (G1 genotype) collected worldwide. In this study, all the sequence data reported from humans related to *E. granulosus s.s*. (G1 genotype), the most common species in humans were screened, and aggregated data were given comparatively. We think that this study can fill the knowledge gaps on the subject. Our findings also represent an important step in future epidemiological, bioecological, vaccine and diagnostic studies that could yield efficient treatments for species/strains.

## Figures and Tables

**Table 1:** Accession numbers of mt-CO1 and mt-ND1 gene fragments of *E. granulosus s.s.* (G1 genotype) isolates used in the study.

mt-CO1			mt-ND1		
Origin	No. of Isolates	Accession Numbers	Origin	No. of Isolates	Accession Numbers
China	43	DQ356874-75-76-77-78-79-80/83, KJ628328-29-30-31-32-33-34-35, AB688602-03-04-05-06-07-08-09-10-11/13-14-15-16-17-18-19, MH050608-09-10-11-12-13-14-15-16-17	Uzbekistan	43	MN696570/72/76-77-78-79-80-81-82-83-84-85-86-87-88-89-90-91-92-93-94-95-96-98-99, MN696600-01-02-03-04/06-07-08-09-10-11-12-13/15/19-20-21-22
Tunisia	13	MG672264-65-66-67-68-69-70-71-72-73-74-75-76	Algeria	20	MG672128, KT316342, KR349038-39-40-41-42-43-44, MG672282/84-85-86-87-88-89-90-91-92-93
Pakistan	12	MK229295-96-97/99, MK229301-02/04/13/15/17-18-19	China	17	AY572548, KJ556993-94, EU072111-12-13-14, MH050620-21-22-23-24-25-26-27-28-29
Mongolia	12	MG672254-55, AB893242-43-44-45-46-47-48-49-50-51	Peru	14	JF946597-98-99, JF946600-01-02-03-04-05-06-07-08-09/24,
Algeria	12	MG672128, MG672283-84-85-86-87-88-89-90-91-92-93	Tunisia	13	MG672264-65-66-67-68-69-70-71-72-73-74-75-76
Turkey	10	EU006783, GU951512-13, MG886833-34-35-36-37-38-39	Iran	9	KT284349, MG672245, JF836800-01-02-03, JF836797-98-99
Iran	9	KR337817, MW350099, MT073987, MG672245, MH025946-47, JQ250810/12/15	Slovenia	6	MT239133-34-35/38/40/42
Russia	9	AB777904/07-08, AB688136-37-38-39-40-41	Spain	4	KU925413-14, MG672129/37
Spain	4	MG672129/37, KU925413-14,	Iraq	3	FJ226756, MN231833-34
Finland	3	MG672132, KY766884, KU925429	Finland	3	MG672132, KY766884, KU925429
India	2	JX854029-30	Mongolia	2	MG672254-55
Morocco	1	EF367266	Poland	2	KT780298- KT780300
Romania	1	MG672138	Romania	1	MG672138
Kazakhstan	1	MG672257	Kazakhstan	1	MG672257
Italy	1	MG672135	Italy	1	MG672135
			Morocco	1	EF367298

**Table 2:** Haplotypes of mt-CO1 sequences of *E. granulosus s.s.* (G1 genotype) and accession numbers of isolates forming groups.

Haplotype Name	No. of Isolate	Accession Numbers
Hap01	2	KJ628335-China, KJ628331-China
Hap02	1	KR337817-Iran
Hap03	79	GU951513-Turkey, GU951512-Turkey, JX854029-India, MG886839-Turkey, MG886838-Turkey, MG886837-Turkey, MG886836-Turkey, MG886835-Turkey, MG886834-Turkey, MG886833-Turkey, DQ356883-China, MW350099-Iran, MT073987-Iran, MK229301-Pakistan, MK229296-Pakistan, MH050617-China, MH050615-China, MH050614-China, MH050612-China, MH050609-China, MH050608-China, MH025946-Iran, KJ628334-China, KJ628333-China, KJ628332-China, KJ628330-China, KJ628329-China, AB688617-China, AB688616-China, AB688614-China, AB688611-China, AB688610-China, AB688609-China, AB688608-China, AB688607-China, AB688603-China, AB688602-China, JQ250815-Iran, AB893250-Mongolia, AB893249-Mongolia, AB893248-Mongolia, AB893247-Mongolia, AB893245-Mongolia, AB893244-Mongolia, AB893243-Mongolia, AB777908-Russia, AB777907-Russia, AB777904-Russia, AB688141-Russia, AB688136-Russia, MG672293-Algeria, MG672292-Algeria, MG672291-Algeria, MG672289-Algeria, MG672288-Algeria, MG672287-Algeria, MG672285-Algeria, MG672284-Algeria, MG672283-Algeria, MG672276-Tunisia, MG672275-Tunisia, MG672274-Tunisia, MG672272-Tunisia, MG672270-Tunisia, MG672269-Tunisia, MG672268-Tunisia, MG672266-Tunisia, MG672265-Tunisia, MG672264-Tunisia, MG672257-Kazakhstan, MG672254-Mongolia, MG672245-Iran, MG672135-Italy, MG672132-Finland, MG672129-Spain, MG672128-Algeria, KY766884-Finland, KU925429-Finland, KU925413-Spain
Hap04	1	EU006783-Turkey
Hap05	2	JX854030-India, MH050610-China
Hap06	2	MK229304-Pakistan, MK229299-Pakistan
Hap07	1	DQ356880-China
Hap08	1	DQ356879-China
Hap09	2	DQ356878-China, AB688604-China
Hap10	12	DQ356877-China, MK229302-Pakistan, MK229297-Pakistan, MH050611-China, JQ250812-Iran, JQ250810-Iran, AB893246-Mongolia, AB688140-Russia, AB688139-Russia, MG672290-Algeria, MG672267-Tunisia, MG672255-Mongolia
Hap11	2	DQ356876-China, DQ356875-China
Hap12	1	DQ356874-China
Hap13	2	MK229319-Pakistan, MK229313-Pakistan
Hap14	3	MK229318-Pakistan, AB688618-China, MG672286-Algeria
Hap15	1	MK229317-Pakistan
Hap16	1	MK229315-Pakistan
Hap17	1	MK229295-Pakistan
Hap18	2	MH050616-China, AB688606-China
Hap19	1	MH050613-China
Hap20	1	EF367266-Morocco
Hap21	1	MH025947-Iran
Hap22	1	KJ628328-China
Hap23	1	AB688619-China
Hap24	1	AB688615-China
Hap25	1	AB688613-China
Hap26	1	AB688605-China
Hap27	1	AB893251-Mongolia
Hap28	1	AB893242-Mongolia
Hap29	1	AB688138-Russia
Hap30	1	AB688137-Russia
Hap31	1	MG672273-Tunisia
Hap32	1	MG672271-Tunisia
Hap33	1	MG672138-Romania
Hap34	2	MG672137-Spain, KU925414-Spain

**Table 3:** Haplotype of mt-ND1 sequences of *E. granulosus s.s.* (G1 genotype) and accession numbers of isolates forming groups.

Haplotype Name	No. of Isolate	Accession Numbers
Hap01	83	KU925413-Spain, EU072111-China, FJ226756-Iraq, KT284349-Iran, JF836803-Iran, JF836802-Iran, JF836801-Iran, JF836799-Iran, JF836797-Iran, MN696622-Uzbekistan, MN696621-Uzbekistan, MN696620-Uzbekistan, MN696619-Uzbekistan, MN696606-Uzbekistan, MN696596-Uzbekistan, MN696591-Uzbekistan, MN696583-Uzbekistan, MN696572-Uzbekistan, MN696570-Uzbekistan, JF946609-Peru, JF946608-Peru, JF946607-Peru, JF946606-Peru, JF946605-Peru, JF946604-Peru, JF946603-Peru, JF946602-Peru, JF946601-Peru, JF946600-Peru, JF946599-Peru, JF946598-Peru, JF946597-Peru, KR349044-Algeria, KR349042-Algeria, KR349038-Algeria, MT239138-Slovenia, MT239133-Slovenia, KT780300-Poland, KT780298-Poland, JF946624-Peru, MH050629-China, MH050628-China, MH050626-China, MH050625-China, MH050622-China, MH050621-China, MH050620-China, KJ556994-China, KJ556993-China, MN231834-Iraq, EF367298-Morocco, MG672293-Algeria, MG672292-Algeria, MG672291-Algeria, MG672289-Algeria, MG672288-Algeria, MG672287-Algeria, MG672285-Algeria, MG672284-Algeria, MG672283-Algeria, MG672276-Tunisia, MG672275-Tunisia, MG672274-Tunisia, MG672273-Tunisia, MG672272-Tunisia, MG672271-Tunisia, MG672270-Tunisia, MG672268-Tunisia, MG672267-Tunisia, MG672266-Tunisia, MG672265-Tunisia, MG672264-Tunisia, MG672257-Kazakhstan, MG672255-Mongolia, MG672254-Mongolia, MG672245-Iran, MG672138-Romania, MG672135-Italy, MG672132-Finland, MG672129-Spain, MG672128-Algeria, KY766884-Finland, KU925429-Finland
Hap02	1	EU072114-China
Hap03	2	EU072113-China, JF836798-Iran
Hap04	2	EU072112-China, AY572548-China
Hap05	15	JF836800-Iran, MN696613-Uzbekistan, MN696612-Uzbekistan, MN696610-Uzbekistan, MN696609-Uzbekistan, MN696607-Uzbekistan, MN696599-Uzbekistan, MN696598-Uzbekistan, MN696590-Uzbekistan, MN696589-Uzbekistan, MN696587-Uzbekistan, MN696585-Uzbekistan, MN696584-Uzbekistan, MN696582-Uzbekistan, MN696581-Uzbekistan
Hap06	1	MN696615-Uzbekistan
Hap07	2	MN696611-Uzbekistan, MN696608-Uzbekistan
Hap08	1	MN696604-Uzbekistan
Hap09	1	MN696603-Uzbekistan
Hap10	1	MN696602-Uzbekistan
Hap11	1	MN696601-Uzbekistan
Hap12	1	MN696600-Uzbekistan
Hap13	1	MN696595-Uzbekistan
Hap14	1	MN696594-Uzbekistan
Hap15	1	MN696593-Uzbekistan
Hap16	1	MN696592-Uzbekistan
Hap17	1	MN696588-Uzbekistan
Hap18	1	MN696586-Uzbekistan
Hap19	1	MN696580-Uzbekistan
Hap20	1	MN696579-Uzbekistan
Hap21	1	MN696578-Uzbekistan
Hap22	1	MN696577-Uzbekistan
Hap23	1	MN696576-Uzbekistan
Hap24	1	KT316342-Algeria
Hap25	1	KR349043-Algeria
Hap26	2	KR349041-Algeria, MG672286-Algeria
Hap27	2	KR349040-Algeria, MG672290-Algeria
Hap28	2	KR349039-Algeria, MG672269-Tunisia
Hap29	1	MT239142-Slovenia
Hap30	1	MT239140-Slovenia
Hap31	1	MT239135-Slovenia
Hap32	1	MT239134-Slovenia
Hap33	1	MH050627-China
Hap34	1	MH050624-China
Hap35	1	MH050623-China
Hap36	1	MN231833-Iraq
Hap37	2	MG672137-Spain KU925414-Spain

**Table 4:** Diversity and neutrality indices obtained using nucleotide data of the mt-CO1 (401 bp) and mt-ND1 (407 bp) genes of *E. granulosus s.s.* (G1 genotype).

	*n*	H	hd ± SD	*π*d ± SD	Tajima’s D	*p* Value	Fu’s Fs	*p* Value	FLD	*p* Value	FLF	*p* Value
mt-CO1	133	34	0.640 ± 0.048	0.00255 ± 0.00031	−2.47269	*. *p* < 0.01	−49.797	0.000	−3.97170	*p* < 0.02	−4.03871	*p* < 0.02
mt-ND1	140	37	0.639 ± 0.047	0.00611 ± 0.00147	−2.80355	*. *p* < 0.001	−31.231	0.000	−8.50154	*p* < 0.02	−7.14019	*p* < 0.02

*n:* Number of isolates, H: number of haplotypes; hd: haplotype diversity; *π*d: nucleotide diversity; SD: standard deviation; FLD: Fu and Li’s D * test statistic; FLF: Fu and Li’s F * test statistic.

## Data Availability

Not applicable.

## Supplementary Materials

The following supporting information can be downloaded at: https://www.mdpi.com/article/10.3390/pathogens11111346/s1, Table S1: Nucleotide variation positions of the mt-CO1 (401 bp) gene among 34 haplotypes analyzed; Table S2: Nucleotide variation positions of the mt-ND1 (407 bp) gene among 37 haplotypes analyzed.
