# Supplementary material for: In Silico Evaluation of the Haplotype Diversity, Phylogenetic Variation and Population Structure of Human E. granulosus sensu stricto (G1 Genotype) Sequences
Source: Pathogens. 2022 Nov 14;11(11):1346. doi: 10.3390/pathogens11111346 (PMC9699212; doi:10.3390/pathogens11111346)
Supplement: Supplementary file 1 [file pathogens-11-01346-s001.zip › pathogens-2010473-supplementary.pdf]

Supplementary Table S1. Nucleotide variation positions of the mt-CO1 (401 bp) gene among 34 haplotypes analyzed

Nucleotid position (n)	7	10	19	20	28	29	60	70	87	91	115	168	178	191	208	211	232	239	250
>MG672129-Spain (Reference sequence)	T	A	A	A	T	A	C	C	C	G	C	T	C	A	T	T	A	T	G
Hap01												A							
Hap02							T	T											
Hap03																			
Hap04			T	C								A							
Hap05														G					
Hap06																			
Hap07													T						
Hap08																			
Hap09														G					
Hap10							T												
Hap11		G																	
Hap12	C							T											
Hap13											T								
Hap14								T											
Hap15											T								
Hap16																			
Hap17																			
Hap18					C														
Hap19																C			
Hap20																		C	
Hap21									T	T									
Hap22																			
Hap23								T			T								

Hap24																			
Hap25																			T
Hap26			G		C														
Hap27																			
Hap28																			
Hap29	C																		
Hap30			G																
Hap31															G				
Hap32						G													
Hap33																	G		
Hap34																			

Nucleotid position (n)	261	262	263	280	316	319	352	353	393	396	397	400
>MG672129-Spain (Reference sequence)	T	T	A	A	G	A	A	G	T	A	T	T
Hap01												
Hap02												
Hap03												
Hap04								A				
Hap05												
Hap06										C		
Hap07												
Hap08					A							
Hap09				G								
Hap10												
Hap11												
Hap12							G					
Hap13												
Hap14												

Hap15					A							
Hap16									G		G	A
Hap17										C	G	
Hap18												
Hap19												
Hap20												
Hap21												
Hap22			G									
Hap23	C											
Hap24						G						
Hap25												
Hap26												
Hap27											C	
Hap28	C											
Hap29												
Hap30												
Hap31												
Hap32												
Hap33												
Hap34		C										

Supplementary Table S2. Nucleotide variation positions of the mt-ND1 (407 bp) gene among 37 haplotypes analyzed

Nucleotid position (n)	2	6	8	10	12	13	14	21	22	25	28	29	34	37	39	42	44	45	51
>KU925413-Spain (Reference sequence)	G	T	C	G	T	C	T	G	T	G	A	T	T	A	G	T	T	T	C
Hap01																			
Hap02											G								
Hap03											G								
Hap04																			
Hap05																			
Hap06																			
Hap07																			
Hap08																			
Hap09																			
Hap10																			
Hap11																			
Hap12																			
Hap13																			
Hap14																			
Hap15	C																		
Hap16								A	C			C						A	
Hap17						A													
Hap18																			
Hap19																			
Hap20																			
Hap21																			A
Hap22																			
Hap23															A		C	C	
Hap24																			
Hap25																			
Hap26						T													
Hap27																			
Hap28																			
Hap29																			
Hap30											G								
Hap31																			
Hap32																			

Hap33																			
Hap34																			
Hap35																			
Hap36		A	A	C	C		C			A	G		A	C	C	C			
Hap37																			

Nucleotid position (n)	52	53	54	55	58	62	67	69	71	73	75	78	79	80	81	82	85	87	88
>KU925413-Spain (Reference sequence)	C	A	A	A	C	G	G	T	G	T	G	T	G	G	T	G	G	G	T
Hap01																			
Hap02																			
Hap03																			
Hap04																			
Hap05																			
Hap06																			
Hap07		G																	
Hap08		G																	
Hap09		G	T			A													
Hap10	A	G	T	T															C
Hap11																			
Hap12																			
Hap13																			
Hap14																			
Hap15																			
Hap16					T														
Hap17																			
Hap18		G	T																
Hap19																			
Hap20		G	T			A													
Hap21		G	T	T		A								T					
Hap22		G	T	T															
Hap23		G	T																
Hap24																			
Hap25																			
Hap26																			
Hap27																			
Hap28																			
Hap29																			
Hap30																			
Hap31																			
Hap32																			

Hap33																			
Hap34																			
Hap35																			
Hap36					T	A	A	C	A	G	A	G	T		C	A	A	A	
Hap37																			

Nucleotid position (n)	89	97	98	104	106	107	110	111	112	129	131	135	143	145	146	148	152	157	161
>KU925413-Spain (Reference sequence)	T	G	C	T	A	T	T	T	T	T	G	T	C	A	G	T	G	C	C
Hap01																			
Hap02																			
Hap03																			
Hap04																			
Hap05																			
Hap06																			
Hap07																			
Hap08																			
Hap09																			
Hap10			T	A		A	A		A		A			T			C		
Hap11												C							
Hap12																			
Hap13				C				G		G			A		A	A		T	A
Hap14								A	A	G			G		A	A			
Hap15																			
Hap16																			
Hap17																			
Hap18																			
Hap19																			
Hap20																			
Hap21	C																		
Hap22																			
Hap23																			
Hap24					G														
Hap25																			
Hap26																			
Hap27					G														
Hap28												C							
Hap29												C							
Hap30												C							
Hap31																			

Hap32		A																	
Hap33			T																
Hap34																			
Hap35																			
Hap36				A															
Hap37																			

Nucleotid position (n)	168	169	178	179	181	182	188	213	222	228	230	234	237	243	244	251	253	255	260
>KU925413-Spain (Reference sequence)	G	T	G	C	G	C	G	T	G	T	A	C	T	A	T	G	T	G	G
Hap01																			
Hap02								C											
Hap03																			
Hap04								C											
Hap05																			
Hap06														G					
Hap07																			
Hap08																			
Hap09																			
Hap10	C						A			A							A	C	A
Hap11																			T
Hap12																			
Hap13		A																	
Hap14																			
Hap15																			
Hap16																			
Hap17																			
Hap18																			
Hap19							C												
Hap20																			
Hap21			C		C		A				C		A	C	C	A			
Hap22																			
Hap23																			
Hap24																			
Hap25																			
Hap26				T															
Hap27									A			T							
Hap28																			
Hap29																			
Hap30																			
Hap31						T													

Hap32																			
Hap33																			
Hap34																			
Hap35																			
Hap36																			
Hap37																			

Nucleotid position (n)	267	272	278	279	281	288	290	298	299	303	305	306	307	309	310	316	317	318	327
>KU925413-Spain (Reference sequence)	T	G	T	T	G	G	C	A	T	T	T	G	G	G	A	T	G	T	T
Hap01																			
Hap02																			
Hap03																			
Hap04																			
Hap05	C																		
Hap06	C																		
Hap07	C																		
Hap08																			
Hap09																			
Hap10		A			A		T		A				A	A		A	A		
Hap11			C		A	C		G		A	A	A							
Hap12																		G	
Hap13																			
Hap14																			
Hap15																			
Hap16																			
Hap17																			
Hap18	C																		
Hap19	C																		
Hap20	C																		
Hap21	C																		
Hap22	C																		
Hap23					C														
Hap24																			
Hap25															G				
Hap26																			
Hap27																			
Hap28																			
Hap29																			C
Hap30																			
Hap31																			

Hap32																			
Hap33																			
Hap34				C															
Hap35																			C
Hap36																			
Hap37																			

Nucleotid position (n)	335	340	345	353	360
>KU925413-Spain (Reference sequence)	G	A	A	T	T
Hap01					
Hap02					
Hap03					
Hap04					
Hap05					
Hap06					
Hap07					
Hap08					
Hap09					
Hap10					
Hap11					
Hap12					
Hap13					
Hap14					
Hap15					
Hap16					
Hap17					
Hap18					
Hap19					
Hap20					
Hap21					
Hap22					
Hap23	A	C	T	G	
Hap24					
Hap25					
Hap26					
Hap27					
Hap28					
Hap29					
Hap30					
Hap31					
Hap32					
Hap33					

Hap34					
Hap35					
Hap36					
Hap37					C
